# Differences in growth within and across the reproductive forms of northern crayfish (*Faxonius virilis*)

**DOI:** 10.1002/ece3.10067

**Published:** 2023-05-17

**Authors:** Doreen Cabrera, Blaine D. Griffen

**Affiliations:** ^1^ Department of Biology Brigham Young University Provo Utah USA

**Keywords:** cyclic dimorphism, growth, life history, reproduction, trade‐offs

## Abstract

Complex life histories are frequently associated with biological trade‐offs, as the use of one trait can decrease the performance of a second trait due to the need to balance competing demands to maximize fitness. Here, we examine growth patterns in invasive adult male northern crayfish (*Faxonius virilis*) that are indicative of a potential trade‐off between energy allocation for body size versus chelae size growth. Northern crayfish undergo cyclic dimorphism, a process characterized by seasonal morphological changes associated with reproductive status. We measured carapace length and chelae length before and after molting and compared these growth increments between the four morphological transitions of the northern crayfish. Consistent with our predictions, reproductive crayfish molting to the non‐reproductive form and non‐reproductive crayfish molting within the non‐reproductive form experienced a larger carapace length growth increment. Reproductive crayfish molting within the reproductive form and non‐reproductive crayfish molting to the reproductive form, on the other hand, experienced a larger growth increment in chelae length. The results of this study support that cyclic dimorphism evolved as a strategy for optimizing energy allocation for body and chelae size growth during discrete periods of reproduction in crayfish with complex life histories.

## Graphical abstract

Complex life histories are frequently associated with biological trade‐offs, as the use of one trait can decrease the performance of a second trait due to the need to balance competing demands to maximize fitness. Here, we examine growth patterns that are indicative of a potential trade‐off between energy allocation for body size versus chelae size growth in a crayfish species that undergoes cyclic dimorphism. The results of this study suggest that cyclic dimorphism evolved as a strategy for optimizing energy allocation for body and chelae size growth during discrete periods of reproductive activity and inactivity in form alternating crayfish.

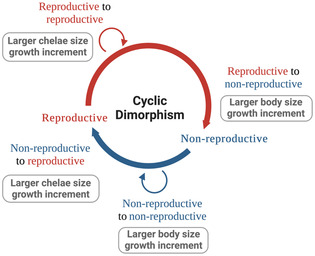

## 1 INTRODUCTION

Biological trade‐offs occur when different traits depend on the same resource (e.g., energy, time, space) causing an increase in the performance of one trait and a decrease in the performance of another trait (Garland, 2014; Stearns, 1989). For example, reproduction is a particularly demanding period, when resources are redirected into tasks necessary to successfully breed (Hendry & Berg, 1999). Such trade‐offs have played a central role in the development of life histories. The relationship between energy allocation for growing in body size and for developing secondary sexual characteristics necessary for reproduction is of particular interest because body size tends to be positively correlated with fecundity (Berglund et al., 1986; Evans, 1982; Sand, 1996). Additionally, growth is a direct investment of future reproduction because the amount of acquired resources scales directly with body size (Peters, 1983), and reproductive effort scales with body size due to allometric constraints (i.e., space and/or capacity; Berrigan, 1991; Kaplan & Salthe, 1979).

When the available energy is subjected to a trade‐off between growing in body size and other traits useful during reproduction, individual differences in growth rate may arise from differences in reproductive status (Dmitriew, 2011). Individual and population‐level resources are an important element driving this energetic trade‐off. For example, variability in internal resources among individuals may skew the relationship between competing traits, since individuals with more energy reserves will be less energetically constrained in resource allocation (Van Noordwijk & de Jong, 1986). Additionally, spatial variation in environmental conditions may promote differences in life history trade‐offs across a species' range (Stearns, 1992). Specifically, negative correlations between energy available for growing in body size versus for other traits are expected to be more pronounced for populations experiencing higher stress (Reznick, 1985). These concepts are widely applicable to many animal taxa; however, there is added complexity when the life history of an organism includes an abrupt ontogenetic change in morphology, physiology, and/or behavior (Cabrera et al., 2021). However, given that available energy is a limiting factor and is constantly under conflicting demands, natural selection should optimize patterns of reproductive development and energy allocation (Gadgil & Bossert, 1970; Williams, 1966).

Animal life cycles are generally described as pertaining to one of two basic modes of development: direct and indirect. Most animals experience indirect development, undergoing metamorphosis that radically alters the phenotypes and function of the larval and adult forms in different ecological settings (Werner, 1988). Many direct developing species experience niche shifts during ontogeny which could effectively be categorized as complex (Werner & Gilliam, 1984). Demands to increase in body size to achieve the adult form and reach reproductive maturity are fundamental for life cycle development (Werner, 1988). However, reproduction in many taxa is frequently interrupted by winter, diapause, or some other seasonal event. For example, form alternation, or cyclic dimorphism, is a unique seasonal event associated with discrete periods of reproduction with associated morphological changes. Form alternating species experience a short non‐breeding stage once a year before returning to the reproductive condition (Payne, 1996).

**FIGURE 1:**
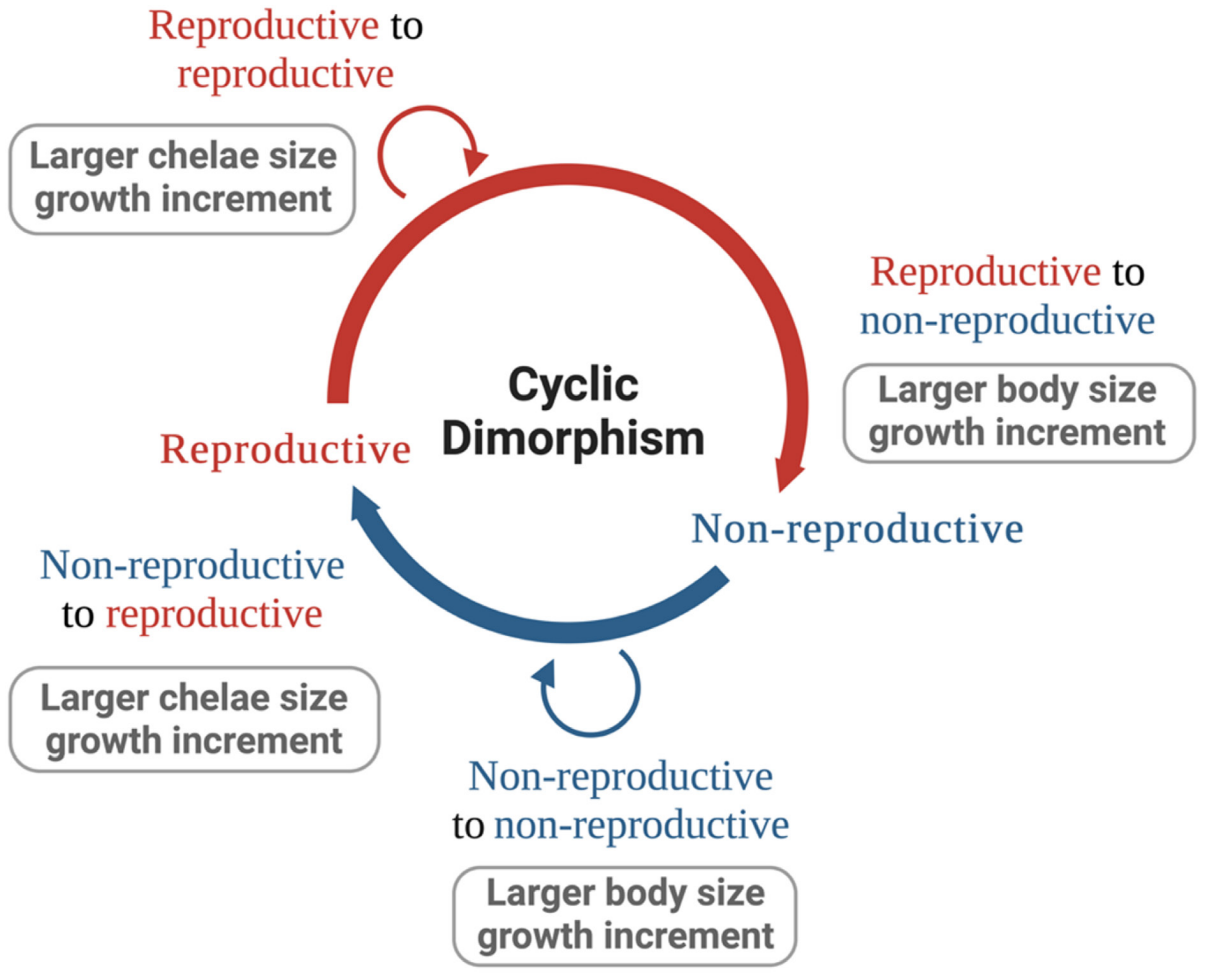
Schematic of cyclic dimorphism in adult male cambarid crayfish. Associated predictions are included in the boxes next to each transition. Form alternating crayfish exhibit four molting transitions within the cycle: reproductive to reproductive, reproductive to non‐reproductive, non‐reproductive to non‐reproductive, and non‐reproductive to reproductive. We note that within‐form molts are less common in nature than molts across forms. Created with BioRender.com.

Adult male crayfish in the cambarid family undergo cyclic dimorphism via seasonal morphological changes between a non‐reproductive morphotype (commonly referred to as form II or FII) and a reproductive morphotype (commonly referred to as form I or FI). The short period of reproductive inactivity occurs in the summer during the intervals between the breeding seasons (Faxon, 1884; Hamr & Berrill, 1985; Mazlum et al., 2007; Payne, 1996; Stein, 1976; Suko, 1958; Figure 1). Mating occurs from July to September (perhaps again in the spring) before crayfish overwinter in the reproductive form (Weagle & Ozburn, 1972). Crayfish subsequently molt to the non‐reproductive form before the start of the summer breeding season (Hamr, 2002). This period of reproductive inactivity is believed to be a growth phase (Huner & Barr, 1991) and a period of regenerating lost limbs (O'Neill et al., 1993). Previously believed to occur only in the Cambaridae family, adult signal crayfish (*Pacifastacus leniusculus*), a crayfish in the Astacidae family, has recently been reported to display morphological patterns consistent with cambarid form alternation (Buřič et al., 2021). Few studies have documented the morphological changes that occur within individual form alternating crayfish after molting (but see Buřič, Kouba, & Kozak, 2010); however, they do not capture all possible form changes. While within‐form changes are less common in nature, they do occur in reproductive males (Buřič, Kouba, & Kozak, 2010).

Crustacean molting is the process of casting off the exoskeleton to permit the expansion of soft tissues and an increase in body size via dimensional increases occurring over intervals (Aiken & Waddy, 1992). However, studying growth in crustaceans is complicated because of the molting process. Linear growth in crustaceans becomes discontinuous, and the loss of the hard exoskeleton during molting makes aging animals ambiguous (Chang et al., 2012). Furthermore, variability in molting frequency and disparities between and within individuals across time and in variable environmental conditions further complicate crustacean growth (Wenner et al., 1974), especially in those that experience complex life histories, such as those exhibiting cyclic dimorphism.

Although energetically costly to produce, crayfish chelae function in prey capture and manipulation, predator defense, inter‐ and intraspecific interactions, and in reproductive activities (Gherardi et al., 2000; Stein, 1976). However, while they are broadly useful, chelae appear to be most important for reproduction and secondary for prey manipulation and defense (Stein, 1976). Male crayfish with larger chelae are more successful during inter‐male competition for mates (e.g., copulation and mate guarding) than similar‐sized males with smaller chelae (Berrill & Arsenault, 1984; Snedden, 1990; Stein, 1976). While chelae size is primarily beneficial during reproductive activities, cambarid crayfish are not always reproductively active. Therefore, continuing to grow larger chelae during the non‐reproductive period would not benefit fecundity and would take energy away from general growth in body size. Energetic trade‐off in growth associated with the discrete periods of reproductive activity and reproductive suspension has been previously determined (Buřič et al., 2021). Specifically, energy should be allocated toward chelae size growth, a trait associated with reproductive activities, when individuals are reproductively active while energy should be allocated to body size growth when reproduction is not viable. For these reasons, we consider here all possible morphological transitions that can occur at a molting event.

Morphological and behavioral differences between forms have been well documented in form alternating crayfish. Besides structural differences in the primary sexual organ (gonopods), reproductive males tend to have larger (Berrill & Arsenault, 1984; Graham et al., 2023; Stein, 1976; Weagle & Ozburn, 1970) and stronger chelae (Graham et al., 2023) than non‐reproductive males. Behaviorally, reproductive males tend to dominate in contests against non‐reproductive males (Berrill & Arsenault, 1984; Guiasu & Dunham, 1998; Martin & Moore, 2010), spend more time in agonistic encounters and less time in shelters than non‐reproductive males (Tierney et al., 2008). Together these well‐established differences between the male forms suggest we should expect differences in the growth increments of form alternating crayfish.

Here we considered the growth increment of the body and chelae sizes of an introduced population of northern crayfish (*Faxonius virilis*) by following individuals across their molting activity. Native to the north‐central United States and south‐central Canada east of the Continental Divide, northern crayfish have been introduced throughout much of the western United States and across the Appalachian region of the eastern United States (Larson et al., 2018), and parts of Europe (Kouba et al., 2014). Previous studies document a variety of body and chelae size ranges in northern crayfish from their native range (Aiken, 1967; Garvey & Stein, 1993; Momot, 1967; Weagle & Ozburn, 1972). While these early studies have made important contributions to understanding growth in the northern crayfish, the role of form alternation in growth patterns has largely been ignored in non‐native populations.

In this study, we investigated the differences in growth increments across and within adult male northern crayfish morphological forms. We measured individual crayfish pre‐molt and post‐molt and compared carapace length and chelae length growth increments across the four transitions: reproductive to reproductive, reproductive to non‐reproductive, non‐reproductive to non‐reproductive, and non‐reproductive to reproductive. In accordance with previous studies, we expected the carapace length growth increment to be larger than the chelae length growth increment for non‐reproductive crayfish molting within the non‐reproductive form and for reproductive crayfish molting to the non‐reproductive form because wielding large chelae is not energetically efficient when crayfish are not capable of reproducing. Furthermore, we expected the chelae length growth increment to be larger than the carapace length growth increment for reproductive crayfish molting within the reproductive form and for non‐reproductive crayfish molting to the reproductive form because chelae size plays an important role in crustacean reproduction.

## 2 MATERIALS AND METHODS

Morphometric data for the present study were derived opportunistically from adult male northern crayfish collected as part of separate experiments that took place over a two‐year period. Crayfish in these separate experiments were held in the laboratory for behavioral measurements that included measuring activity (*n* = 156) and aggression (*n* = 84). Depending on the nature of the experiment, crayfish were held in the laboratory for various lengths of time (3–6 weeks). As a result, long‐term housing allowed us to capture morphological measurements before and after molting events as they occurred. Therefore, measurements for the present study were opportunistically collected from 33 of these crayfish that happened to molt while being held in the lab, as follows: 12 in 2019 and 21 in 2020. All crayfish originated from a non‐native population located at Strawberry Reservoir, Wasatch County, Utah (40°09′55.95″ N, 111°11′16.781″ W). Introductions of northern crayfish in this region are presumed to have been initiated by management agencies as a source of forage for non‐native sportfish (Johnson, 1986). Because crayfish in this study were collected for separate experiments, crayfish were collected at different dates throughout the late spring and summer months (June–September) where surface temperatures ranged from 15.9 to 20.8°C. Upon capture by hand (*n* = 27) and with bait (*n* = 6), crayfish were transported to a laboratory at Brigham Young University. Animals were housed individually in plastic aquaria (22.9 × 15.2 × 16.5 cm, L × W × H) filled with dechlorinated tap water and were held under a 16 h:8 h light : dark cycle. Temperatures in individual plastic aquaria ranged from 17.8 to 20.7°C. Each container was continually aerated and supplied with a small piece of PVC shelter. Crayfish were fed twice a week ad libitum with commercial sinking wafers (Hikari).

Morphometric measurements were recorded upon arrival to the laboratory and at least one‐week post‐molting to allow for the hardening of the exoskeleton. All crayfish had two fully intact chelae with no obvious indication of regeneration. We measured carapace length, defined as the tip of the rostral apex to the posterior median edge of the cephalothorax (Brewis & Bowler, 1982; Garvey & Stein, 1993), to the nearest 0.1 mm with vernier calipers. We also measured the right chelae length from the carpal joint to the distal tip of the propodus (Garvey & Stein, 1993) in the same manner.

We identified reproductive status (reproductive versus non‐reproductive) upon collection and post‐molting by visual inspection of the swimmerets (pleopods; Huner & Barr, 1991). Reproductive males have more calcified and enlarged copulatory pleopods (gonopods) than non‐reproductive males (Huner & Barr, 1991; Stein et al., 1977). Details of the sample size, carapace length, and chelae length size range for each transition are summarized in Table 1. Northern crayfish mature within the first or second year between 23 and 27 mm carapace length (Hamr, 2002). Based on our crayfish collections at Strawberry Reservoir conducted between 2019–2022, the smallest reproductively active male northern crayfish that we collected was 27.8 mm carapace length. Three of the non‐reproductive crayfish that remained non‐reproductive post‐molting fall on the lower end of the range suggested by Hamr (2002), however, we were interested in capturing the growth increments between the four possible morphological transitions. Considering that six of the non‐reproductive crayfish that remained non‐reproductive post‐molting fell above the range, we did not exclude the three smaller non‐reproductive crayfish from the analysis.

**TABLE 1:** Carapace length and chelae length size ranges before molting and mean ± standard deviation before and after molting in male northern crayfish (*Faxonius virilis*). Data are presented in millimeters (mm). Sample sizes are included below each transition.

	Before molting size range	Before molting mean **±** SD	After molting mean **±** SD
Carapace length (mm)			
Reproductive to reproductive (*n* = 4)	37.3–53.2	43.6 ± 3.5	45.0 ± 3.4
Reproductive to non‐reproductive (*n* = 12)	29.0–46.7	36.2 ± 1.8	39.0 ± 1.7
Non‐reproductive to non‐reproductive (*n* = 9)	24.9–45.7	32.2 ± 2.5	34.7 ± 2.5
Non‐reproductive to reproductive (*n* = 8)	30.0–47.7	38.0 ± 2.3	40.0 ± 2.2
Chelae length (mm)			
Reproductive to reproductive (*n* = 4)	26.5–43.5	33.6 ± 3.6	37.9 ± 3.6
Reproductive to non‐reproductive (*n* = 12)	15.7–37.9	26.8 ± 2.3	27.8 ± 2.1
Non‐reproductive to non‐reproductive (*n* = 9)	13.0–37.6	21.0 ± 3.0	23.0 ± 2.7
Non‐reproductive to reproductive (*n* = 8)	18.3–40.9	25.7 ± 2.7	29.3 ± 2.7

### 2.1 Statistical analysis

To determine whether there were differences in the carapace length and chelae length growth increments between the four morphological transitions, we ran separate ANCOVA models to control for pre‐molt carapace length and chelae length. We square‐root transformed the carapace length and chelae length growth increments to normalize the data prior to fitting the models. We fit an ANCOVA with the carapace length growth increment as the response variable, morphological transition as a predictor variable, and pre‐molt carapace length as the covariate. Next, we fit an ANCOVA with the chelae length growth increment as the response variable, morphological transition as a predictor variable, and pre‐molt chelae length as the covariate. For both these analyses, we initially included the interaction term between morphological transition and pre‐molt sizes. However, the interaction term was not significant in either analysis (carapace length: *p* = .403; chelae length: *p* = .681) and so was excluded from the models. Following each ANCOVA, we performed a Tukey's Test for multiple comparisons using the *multcomp* package (Hothorn et al., 2008) to identify which specific morphological transitions yielded significant differences in carapace length and chelae length growth increments, respectively. We report the means for carapace length and chelae length for each transition. Statistical analyses were performed in R Software Version 4.2.1 (R Core Team, 2022).

## 3 RESULTS

**FIGURE 2:**
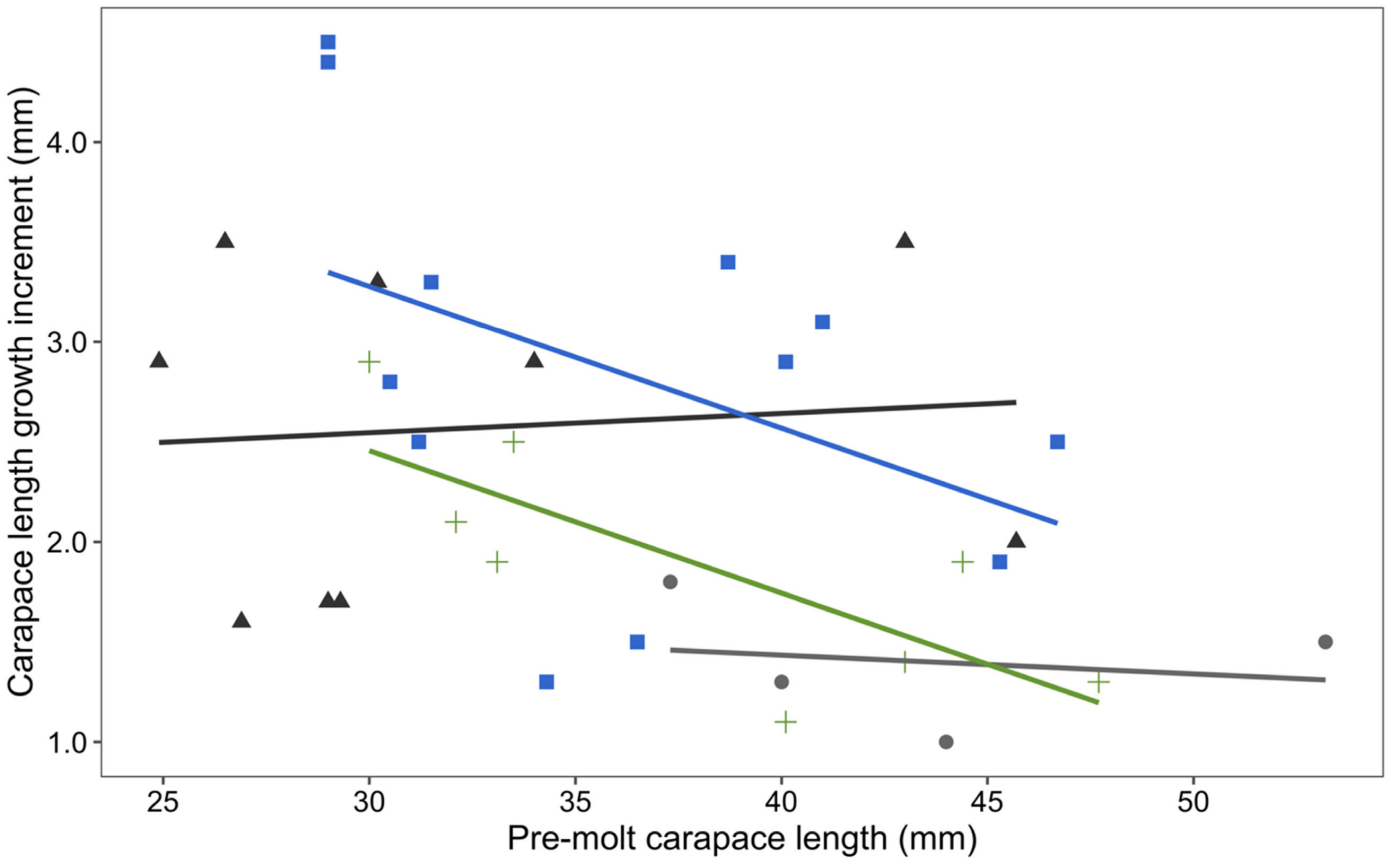
Relationship between the carapace length growth increment (mm) and pre‐molt carapace length (mm) in male northern crayfish (*Faxonius virilis*). Circles (gray) = reproductive to reproductive; squares (blue) = reproductive to non‐reproductive; triangles (black) = non‐reproductive to non‐reproductive; crosses (green) = non‐reproductive to reproductive. Colored lines represent the linear relationship between the carapace length growth increment and pre‐molt carapace length for each corresponding transition.

**FIGURE 3:**
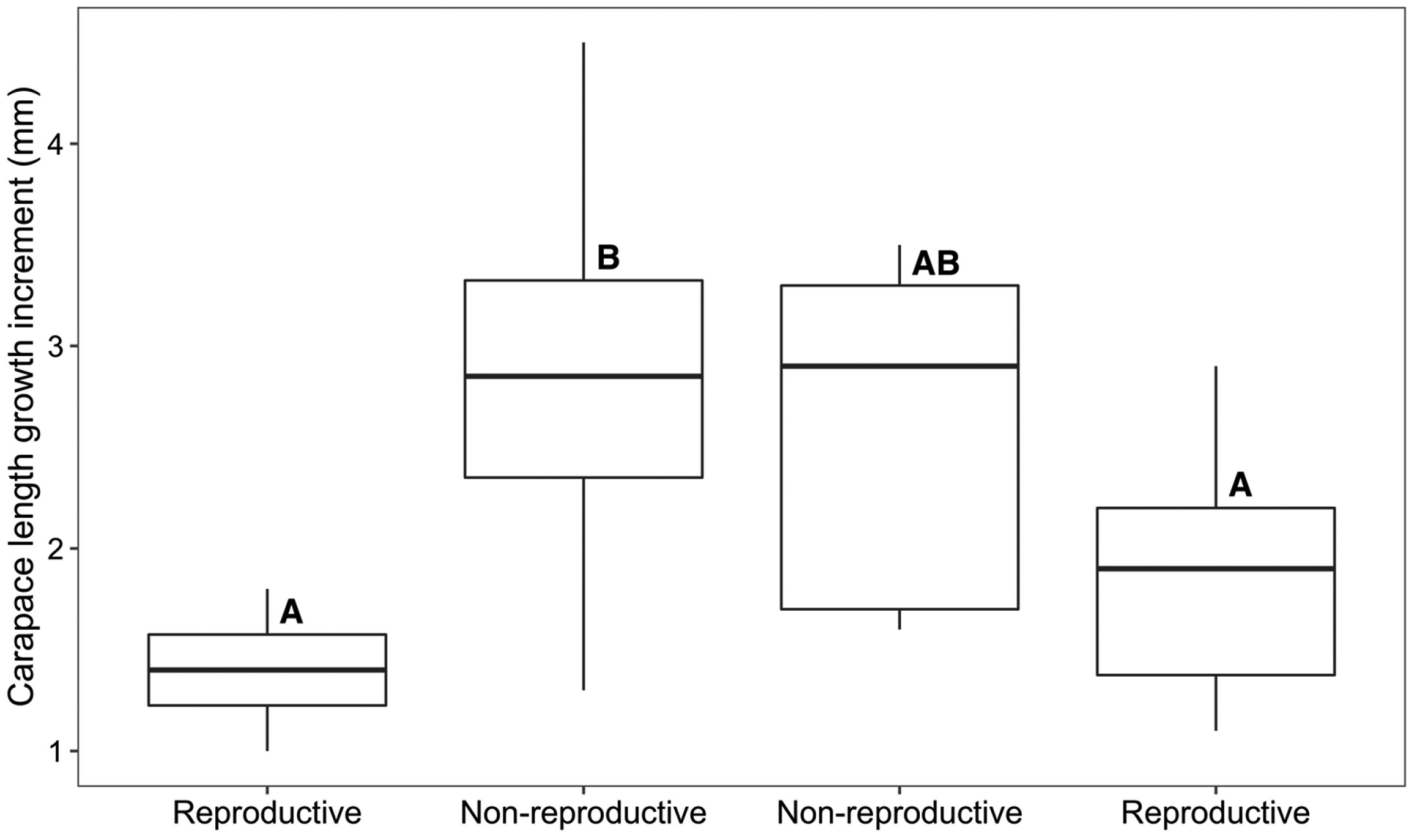
Carapace length growth increments (mm) for the four morphological transitions of the male north crayfish (*Faxonius virilis*). Labels on the x‐axis represent the post‐molt forms. In order from left to right: reproductive to reproductive, reproductive to non‐reproductive, non‐reproductive to non‐reproductive, and non‐reproductive to reproductive. Thick horizontal lines within the boxes show the median, each box encompasses the first and third quartiles of data, and whiskers encompass 95% of the data. The capital letters above the boxes indicate statistical significance.

We found that the carapace length growth increment differed across morphological transitions (*F*_3,28_ = 3.093, *p* = .043), but was not influenced by pre‐molt carapace length (*F*_1,28_ = 2.768, *p* = .107). The carapace length growth increment for reproductive crayfish molting to the non‐reproductive form (mean ± SD; 2.8 ± 0.3 mm) was larger than for reproductive crayfish molting within the reproductive form (mean ± SD; 1.4 ± 0.2 mm), though this difference was only moderately significant (*p* = .071; Figures 2 and 3). The carapace length growth increment for reproductive crayfish molting to the non‐reproductive form given above was also larger than for non‐reproductive crayfish molting to the reproductive form (mean ± SD; 1.9 ± 0.2 mm), but again, this difference was only moderately significant (*p* = .095; Figures 2 and 3). The mean carapace length growth increment for non‐reproductive crayfish molting within the non‐reproductive form was 2.6 ± 0.3 mm. The mean ± SD for carapace length before and after molting is summarized in Table 1.

**FIGURE 4:**
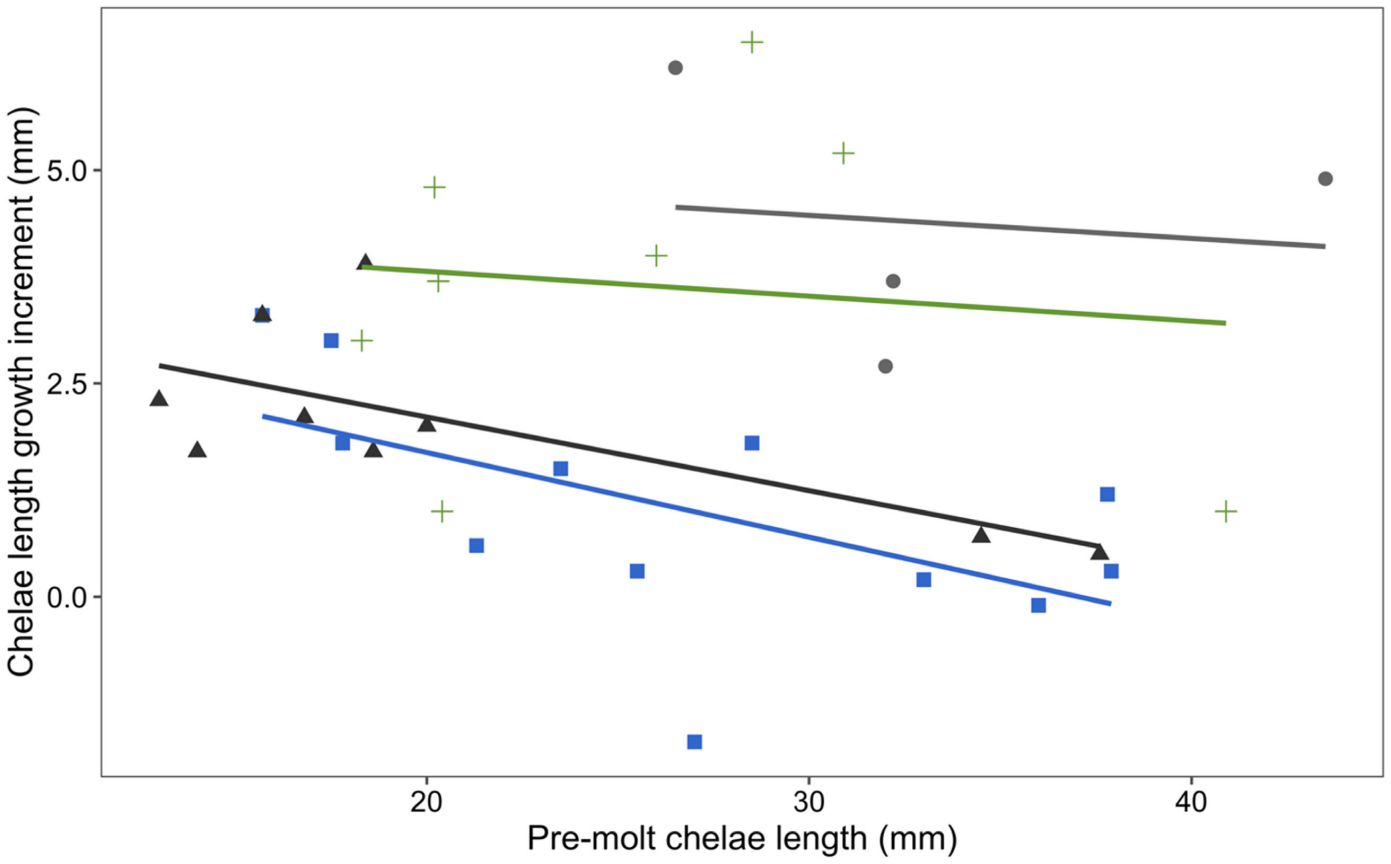
Relationship between the chelae length growth increment (mm) and pre‐molt chelae length (mm) in male northern crayfish (*Faxonius virilis*). Circles (gray) = reproductive to reproductive; squares (blue) = reproductive to non‐reproductive; triangles (black) = non‐reproductive to non‐reproductive; crosses (green) = non‐reproductive to reproductive. Colored lines represent the linear relationship between the chelae length growth increment and pre‐molt carapace length for each corresponding transition.

**FIGURE 5:**
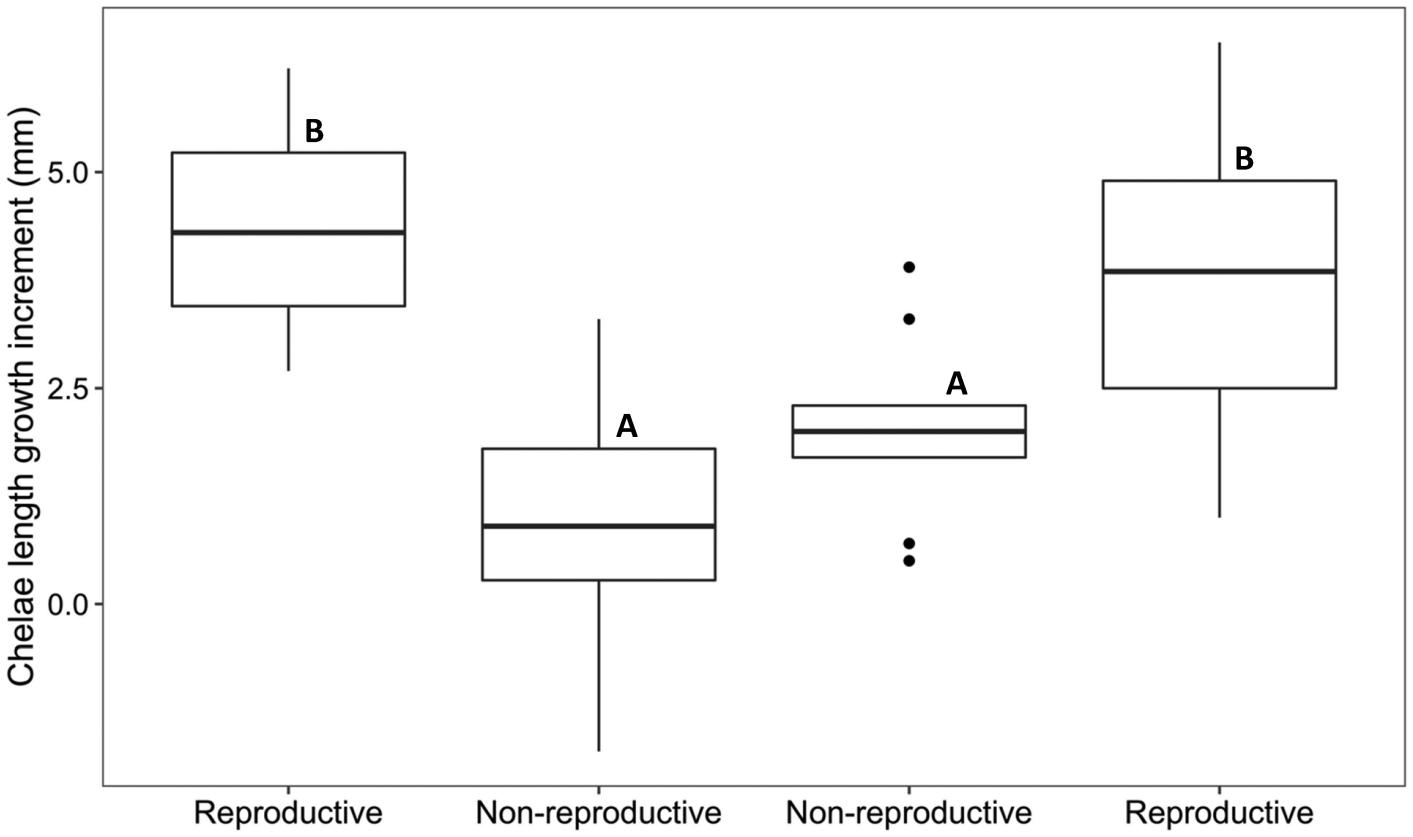
Chelae length growth increments (mm) for the four morphological transitions of the male north crayfish (*Faxonius virilis*). Labels on the x‐axis represent the post‐molt forms. In order from left to right: reproductive to reproductive, reproductive to non‐reproductive, non‐reproductive to non‐reproductive, and non‐reproductive to reproductive. Thick horizontal lines within the boxes show the median, each box encompasses the first and third quartiles of data, whiskers encompass 95% of the data, and circles represent outliers that fall outside of this range. The capital letters above the boxes indicate statistical significance.

We found that the chelae length growth increment differed across morphological transitions (*F*_3,26_ = 9.744, *p* < .001) and was influenced by pre‐molt chelae length (*F*_1,26_ = 8.832, *p* = .006). The chelae length growth increment for non‐reproductive crayfish molting within the non‐reproductive form (mean ± SD; 2.0 ± 0.4 mm) was smaller than for reproductive crayfish molting within the reproductive form (mean ± SD; 4.4 ± 0.8 mm; *p* = .004; Figures 4, 5). The chelae length growth increment for reproductive crayfish molting to the non‐reproductive form (mean ± SD; 1.0 ± 0.4 mm) was smaller than for reproductive crayfish molting within the reproductive form (*p* < .001; Figures 4 and 5). The chelae length growth increment for non‐reproductive crayfish molting to the reproductive form (mean ± SD; 3.7 ± 0.7 mm) was larger than both reproductive crayfish molting to the non‐reproductive form (*p* = .005; Figures 4 and 5) and for non‐reproductive crayfish molting within the non‐reproductive form (*p* = .036; Figures 4 and 5). The mean ± SD for chelae length before and after molting is summarized in Table 1.

## 4 DISCUSSION

We found moderate differences in carapace length growth increments but clear evidence of differences in chelae length growth increments between the male northern crayfish's reproductive and non‐reproductive morphological forms. The carapace length growth increment for reproductive crayfish molting to the non‐reproductive form was larger than reproductive crayfish molting within the reproductive form and non‐reproductive crayfish molting to the reproductive form. This is in line with a previous study that suggested that when in captivity, non‐reproductive males can gain body size and regenerate lost chelae to make them more competitive with intact males (O'Neill et al., 1993). Chelae length growth increments were largest for crayfish molting within the reproductive form and for those transitioning from the non‐reproductive form to the reproductive form. This is consistent with what we expected for reproductively active crayfish since, in many decapod crustaceans, chelae size is essential for mating success (Garvey & Stein, 1993; Snedden, 1990; Stein, 1976). Together, these results support previous evidence of trade‐offs between energy allocation for body size versus chelae size growth (Buřič et al., 2021; Guiasu & Dunham, 1998; Stein, 1976), unique to crayfish experiencing cyclical patterns in reproductive morphology.

Selective forces have offset the energetic costs associated with reproducing and carrying large chelae by undergoing a relatively brief period of reproductive suspension with a simultaneous reduction in chelae size and an increase in carapace size. This is important to note because a more robust carapace allows for more space for the hepatopancreas, the primary energy storage organ in crustaceans (O'Connor & Gilbert, 1968). The hepatopancreas not only serves to finance growth in crustaceans but also for egg production via the ovaries (Lovrich et al., 2005) in the case of female crayfish. Form alternation in females has received less attention than in males; however, it has been detected in some species (Buřič, Kouba, & Kozák, 2010; Wetzel, 2002).

We found the chelae length growth increment to be largest for non‐reproductive to reproductive form molts and for within reproductive form molts, in line with our predictions. Most male cambarid crayfish have larger chelae than females (Bovbjerg, 1953; Gu et al., 1994; Weagle & Ozburn, 1970), thus selection for large chelae is greater for males than for females (Stein, 1976). Large chelae in males confer an advantage in interactions with large fecund females and contribute to successfully accessing females against competing males (Berrill & Arsenault, 1984; Snedden, 1990). Inter‐form contests between reproductive and non‐reproductive males suggest that reproductive males tend to be more dominant and initiate contests more frequently than non‐reproductive males (Guiasu & Dunham, 1998). Non‐breeding males in our study experienced a reduction in chelae size growth because they are not engaging in reproductive activities. When reproduction is suspended, less energy would be spent on developing and maintaining large cumbersome chelae (Stein, 1976). As such, cyclic dimorphism evolved as a strategy to optimize growth and reproductive performance in form alternating crayfish.

Adult male cambarid crayfish in their native range generally molt in mid‐June from the reproductive form to the non‐reproductive form (Capelli & Magnuson, 1983); however, northern crayfish from our study site molt to the non‐reproductive form mid‐to‐late July in the wild, possibly reflecting latitudinal and/or species‐specific variation. We have collected northern crayfish throughout the breeding season from the same study site from 2019 to 2022 for separate studies. The smallest reproductively active male from this site collected to date was 27.8 mm in carapace length, consistent with the previously determined maturity range (Hamr, 2002). Regardless of range, non‐reproductive males tend to have smaller chelae than reproductive males (Stein, 1976) and our results suggest that the chelae length growth increment is indeed smaller for reproductive crayfish molting to the non‐reproductive form and for non‐reproductive to non‐reproductive transitions.

Body size growth of northern crayfish from their native range has been measured in several studies. Although in this study we did not explicitly compare growth patterns between native and introduced populations of northern crayfish, we made an effort to understand the implications that introductions have had on the life history of this species. A comparison of the carapace length ranges of northern crayfish from three native areas suggests some similarities. The following are previously recorded carapace length ranges for adult males: 36–42 mm in Alberta, Canada (Aiken, 1967); 36–42 mm in Michigan, United States (Momot, 1967), and 32–44 mm for male crayfish in N.W. Ontario, Canada (Weagle & Ozburn, 1972). However, these studies did not consider reproductive form in their measurements. Carapace length ranges for crayfish at our site tended to be larger, particularly for reproductive crayfish molting within the reproductive form (37.3–53.2 mm). Additionally, the carapace length range that we recorded here is the opposite of what we would expect according to Bergman's rule which suggests that large‐bodied species tend to live at higher latitudes than their smaller‐bodied relatives (Blackburn et al., 1999). We recognize that we had a relatively small number of molting events per each morphological transition, however, the results that we obtained suggest that other factors such as interspecific competition or invasion success could play a larger role than latitude in shaping the life history of this species.

Introduced species often display increased body size within their non‐native ranges relative to their native range, including in invertebrates (Grosholz & Ruiz, 2003; Pintor & Sih, 2009). It is possible that introduced populations are experiencing a selective filter resulting in systematic differences in life history traits before versus after introduction (Pintor & Sih, 2009), an indication of predator release (Colautti et al., 2004; Keane & Crawley, 2002), less intense intraspecific interactions (Snyder & Evans, 2006), or differences in abilities to exploit resources (Mooney & Cleland, 2001; Pintor, 2007; Snyder & Evans, 2006). Additionally, populations living in high‐predator locations have been shown to reach sexual maturity earlier and at smaller body sizes and give birth to smaller offspring than individuals living in low‐predation environments (Reznick, 1982). Northern crayfish have been implicated in causing the displacement of native crayfish (Larson et al., 2018) as well as being a competitor with native fish for food resources (Carpenter, 2005). Larger body sizes and differences in growth increments could be driving these negative interactions between northern crayfish and competing species in their invaded range. Updated comparisons of body size in native populations versus in introduced population of northern crayfish are needed to assess whether the predictions we have made here are contributing to its competitive advantage over native crayfish species.

Previous studies that have observed the different forms of crayfish at distinct times have assumed that crayfish do not molt within forms (Berrill, 1978; Hamr & Berrill, 1985). We note that these studies did not collect crayfish for long‐term observation and determining whether crayfish molt within forms requires repeated observations of single individuals. We have shown that within form molting can occur in the northern crayfish. Evidence exists of form alternating crayfish molting once in captivity without a form alternation, but this was likely due to larger initial size, than those that molted between forms (Buřič, Kouba, & Kozak, 2010). Additional data are needed to determine the extent to which within‐form molting occurs in the wild, and whether this possibly differs across populations (e.g., native vs. invasive) or is influenced by captivity.

The findings in this study moderately confirmed our predicted growth increment for body size. Still, they showed clear support for chelae size growth predictions associated with the morphological transitions of the northern crayfish. These observations are consistent with the biological trade‐off that we expected between energy allocation for body size and chelae size from a species that experience discrete periods of breeding activity and inactivity. Our results are an important contribution to life history theory, particularly in organisms that are characterized by complex developmental traits. We have shown in this study that cyclic dimorphism may have evolved to maximize chelae size for the discrete reproductive period, while simultaneously increasing body size. Ultimately, such complex processes are necessary means for meeting the fitness requirements that lead to species' success.

## AUTHOR CONTRIBUTIONS

**Doreen Cabrera:** Conceptualization (equal); data curation (lead); methodology (lead); formal analysis (equal); writing – original draft (lead); writing – review and editing (equal). **Blaine D. Griffen:** Conceptualization (equal); formal analysis (equal); supervision (lead); writing – review and editing (equal).

## CONFLICT OF INTEREST STATEMENT

The authors declare no competing interests.

## Data Availability

Data are available in the Dryad Digital Repository https://doi.org/10.5061/dryad.15dv41p2j (Cabrera & Griffen, 2023).

## References

[ece310067-bib-0001] Aiken, D. E. (1967). Environmental regulation of molting and reproduction in the crayfish *Orconectes virilis* (Hagen) in Alberta. (Doctoral dissertation) University of Alberta.

[ece310067-bib-0002] Aiken, D. E. , & Waddy, S. L. (1992). The growth process in crayfish. Reviews in Aquatic Sciences, 6(3,4), 335–381.

[ece310067-bib-0003] Berglund, A. , Rosenqvist, G. , & Svensson, I. (1986). Mate choice, fecundity and sexual dimorphism in two pipefish species (Syngnathidae). Behavioral Ecology and Sociobiology, 19(4), 301–307. 10.1007/BF00300646

[ece310067-bib-0004] Berrigan, D. (1991). The allometry of egg size and number in insects. Oikos, 60(3), 313–321. 10.2307/3545073

[ece310067-bib-0005] Berrill, M. (1978). Distribution and ecology of crayfish in the Kawartha Lakes region of southern Ontario. Canadian Journal of Zoology, 56(2), 166–177. 10.1139/z78-026

[ece310067-bib-0006] Berrill, M. , & Arsenault, M. (1984). The breeding behaviour of a northern temperate orconectid crayfish, *Orconectes rusticus* . Animal Behaviour, 32(2), 333–339. 10.1016/S0003-3472(84)80265-1

[ece310067-bib-0007] Blackburn, T. M. , Gaston, K. J. , & Loder, N. (1999). Geographic gradients in body size: A clarification of Bergmann's rule. Diversity and Distributions, 5(4), 165–174. 10.1046/j.1472-4642.1999.00046.x

[ece310067-bib-0008] Bovbjerg, R. V. (1953). Dominance order in the crayfish *Orconectes virilis* (Hagen). Physiological Zoology, 26(2), 173–178. 10.1086/physzool.26.2.30154514

[ece310067-bib-0009] Brewis, J. M. , & Bowler, K. (1982). The growth of the freshwater crayfish *Austropotamobius pallipes* in Northumbria. Freshwater Biology, 12(2), 187–200. 10.1111/j.1365-2427.1982.tb00613.x

[ece310067-bib-0010] Buřič, M. , Haubrock, P. J. , Veselý, L. , Kozák, P. , & Kouba, A. (2021). Effective investments due to seasonal morphological changes? Possible reasons and consequences of allometric growth and reproduction in adult signal crayfish (*Pacifastacus leniusculus*). Canadian Journal of Zoology, 99(2), 85–96. 10.1139/cjz-2020-0155

[ece310067-bib-0011] Buřič, M. , Kouba, A. , & Kozak, P. (2010). Molting and growth in relation to form alternations in the male spiny‐cheek crayfish *Orconectes limosus* . Zoological Studies, 49(1), 28–38.

[ece310067-bib-0012] Buřič, M. , Kouba, A. , & Kozák, P. (2010). Intra‐sex dimorphism in crayfish females. Zoology, 113(5), 301–307. 10.1016/j.zool.2010.06.001 20932733

[ece310067-bib-0013] Cabrera, D. , & Griffen, B. D. (2023). Data for: Northern crayfish form alternation morphometrics. Dryad, Dataset. 10.5061/dryad.15dv41p2j

[ece310067-bib-0014] Cabrera, D. , Nilsson, J. R. , & Griffen, B. D. (2021). The development of animal personality across ontogeny: A cross‐species review. Animal Behaviour, 173, 137–144. 10.1016/j.anbehav.2021.01.003

[ece310067-bib-0015] Capelli, G. M. , & Magnuson, J. J. (1983). Morphoedaphic and biogeographic analysis of crayfish distribution in northern Wisconsin. Journal of Crustacean Biology, 3(4), 548–564. 10.2307/1547950

[ece310067-bib-0016] Carpenter, J. (2005). Competition for food between an introduced crayfish and two fishes endemic to the Colorado River basin. Environmental Biology of Fishes, 72(3), 335–342. 10.1007/s10641-004-2588-z

[ece310067-bib-0017] Chang, Y. J. , Sun, C. L. , Chen, Y. , & Yeh, S. Z. (2012). Modelling the growth of crustacean species. Reviews in Fish Biology and Fisheries, 22(1), 157–187. 10.1007/s11160-011-9228-4

[ece310067-bib-0018] Colautti, R. I. , Ricciardi, A. , Grigorovich, I. A. , & MacIsaac, H. J. (2004). Is invasion success explained by the enemy release hypothesis? Ecology Letters, 7(8), 721–733. 10.1111/j.1461-0248.2004.00616.x

[ece310067-bib-0019] Dmitriew, C. M. (2011). The evolution of growth trajectories: What limits growth rate? Biological Reviews, 86(1), 97–116. 10.1111/j.1469-185X.2010.00136.x 20394607

[ece310067-bib-0020] Evans, E. W. (1982). Consequences of body size for fecundity in the predatory stinkbug, *Podisus maculiventris* (Hemiptera: Pentatomidae). Annals of the Entomological Society of America, 75(4), 418–420. 10.1093/aesa/75.4.418

[ece310067-bib-0021] Faxon, W. (1884). ART. VIII. On the so‐called dimorphism in the genus *Cambarus* . American Journal of Science (1880–1910), 27(157–162), 42. 10.2475/ajs.s3-27.157.42

[ece310067-bib-0022] Gadgil, M. , & Bossert, W. H. (1970). Life historical consequences of natural selection. The American Naturalist, 104(935), 1–24. 10.1086/282637

[ece310067-bib-0023] Garland, T. (2014). Trade‐offs. Current Biology, 24(2), R60–R61. 10.1016/j.cub.2013.11.036 24456973

[ece310067-bib-0024] Garvey, J. E. , & Stein, R. A. (1993). Evaluating how chela size influences the invasion potential of an introduced crayfish (*Orconectes rusticus*). American Midland Naturalist, 129(1), 172–181. 10.2307/2426446

[ece310067-bib-0025] Gherardi, F. , Acquistapace, P. , & Barbaresi, S. (2000). The significance of chelae in the agonistic behaviour of the white‐clawed crayfish, *Austropotamobius pallipes* . Marine and Freshwater Behaviour and Physiology, 33(3), 187–200. 10.1080/10236240009387090

[ece310067-bib-0026] Graham, Z. A. , Stubbs, M. B. , & Loughman, Z. J. (2023). Male crayfish (*Faxonius obscurus*, Decapoda: Cambaridae) claws are longer and stronger yet comparable in shape during the reproductive season. Biological Journal of the Linnean Society, 139(1), 57–69. 10.1093/biolinnean/blad018

[ece310067-bib-0027] Grosholz, E. D. , & Ruiz, G. M. (2003). Biological invasions drive size increases in marine and estuarine invertebrates. Ecology Letters, 6(8), 700–705. 10.1046/j.1461-0248.2003.00495.x

[ece310067-bib-0028] Gu, H. , Mather, P. B. , & Capra, M. F. (1994). The relative growth of chelipeds and abdomen and muscle production in male and female red claw crayfish, *Cherax* *quadricarinatus* von Martens. Aquaculture, 123(3–4), 249–257. 10.1016/0044-8486(94)90063-9

[ece310067-bib-0029] Guiasu, R. C. , & Dunham, D. W. (1998). Inter‐form agonistic contests in male crayfishes, *Cambarus robustus* (Decapoda, Cambaridae). Invertebrate Biology, 117(2), 144–154. 10.2307/3226966

[ece310067-bib-0030] Hamr, P. (2002). Orconectes. In D. M. Holdich (Ed.), Biology of freshwater crayfish (Vol. 702, pp. 585–608). Blackwell Science.

[ece310067-bib-0031] Hamr, P. , & Berrill, M. (1985). The life histories of north‐temperate populations of the crayfish *Cambarus robustus* and *Cambarus bartoni* . Canadian Journal of Zoology, 63(10), 2313–2322. 10.1139/z85-343

[ece310067-bib-0032] Hendry, A. P. , & Berg, O. K. (1999). Secondary sexual characters, energy use, senescence, and the cost of reproduction in sockeye salmon. Canadian Journal of Zoology, 77(11), 1663–1675. 10.1139/z99-158

[ece310067-bib-0033] Hothorn, T. , Bretz, F. , & Westfall, P. (2008). Simultaneous inference in general parametric models. Biometrical Journal, 50(3), 346–363. 10.1002/bimj.200810425 18481363

[ece310067-bib-0034] Huner, J. V. , & Barr, J. E. (1991). Red swamp crawfish: Biology and exploitation (3rd ed.). Louisiana Sea Grant College Program.

[ece310067-bib-0035] Johnson, J. E. (1986). Inventory of Utah crayfish with notes on current distribution. The Great Basin Naturalist, 46(4), 625–631.

[ece310067-bib-0036] Kaplan, R. H. , & Salthe, S. N. (1979). The allometry of reproduction: An empirical view in salamanders. The American Naturalist, 113(5), 671–689. 10.1086/283425

[ece310067-bib-0037] Keane, R. M. , & Crawley, M. J. (2002). Exotic plant invasions and the enemy release hypothesis. Trends in Ecology & Evolution, 17(4), 164–170. 10.1016/S0169-5347(02)02499-0

[ece310067-bib-0038] Kouba, A. , Petrusek, A. , & Kozák, P. (2014). Continental‐wide distribution of crayfish species in Europe: Update and maps. Knowledge and Management of Aquatic Ecosystems, 413(5), 1–31. 10.1051/kmae/2014007

[ece310067-bib-0039] Larson, E. R. , Egly, R. M. , & Williams, B. W. (2018). New records of the non‐native virile crayfish *Faxonius virilis* (Hagen, 1870) from the upper Snake River drainage and northern Bonneville Basin of the western United States. BioInvasions Records, 7(2), 177–183. 10.3391/bir.2018.7.2.10

[ece310067-bib-0040] Lovrich, G. A. , Romero, M. C. , Tapella, F. , & Thatje, S. (2005). Distribution, reproductive and energetic conditions of decapod crustaceans along the scotia arc (Southern Ocean). Scientia Marina, 69(2), 183–193. 10.3989/scimar.2005.69s2183

[ece310067-bib-0041] Martin, A. L., III , & Moore, P. (2010). The influence of reproductive state on the agonistic interactions between male and female crayfish (*Orconectes rusticus*). Behaviour, 147(10), 1309–1325. 10.1163/000579510X520989

[ece310067-bib-0042] Mazlum, Y. , Can, M. F. , & Eversole, A. G. (2007). Morphometric relationship of length‐weight and chelae length‐width of eastern white river crayfish (*Procambarus acutus acutus*, Girard, 1852), under culture conditions. Journal of Applied Ichthyology, 23(5), 616–620. 10.1111/j.1439-0426.2007.01015.x

[ece310067-bib-0043] Momot, W. T. (1967). Population dynamics and productivity of the crayfish, *Orconectes virilis*, in a marl lake. American Midland Naturalist, 78(1), 55–81. 10.2307/2423370

[ece310067-bib-0044] Mooney, H. A. , & Cleland, E. E. (2001). The evolutionary impact of invasive species. Proceedings of the National Academy of Sciences, 98(10), 5446–5451. 10.1073/pnas.091093398 PMC33232 11344292

[ece310067-bib-0045] O'Connor, J. D. , & Gilbert, L. I. (1968). Aspects of lipid metabolism in crustaceans. American Zoologist, 8(3), 529–539. 10.1093/icb/8.3.529 5699279

[ece310067-bib-0046] O'Neill, D. J. , French, D. P. , Rebach, S. , & Handwerker, T. S. (1993). Chelae removal alters attainment of sexual maturity in male *Procambarus clarkii* (Girard) and mortality in groups. Freshwater Crayfish, 9, 38–49.

[ece310067-bib-0047] Payne, J. F. (1996). Adaptive success within the cambarid life cycle. Freshwater Crayfish, 11, 1–12.

[ece310067-bib-0048] Peters, R. H. (1983). The ecological implications of body size (Vol. 2). Cambridge University Press. 10.1017/CBO9780511608551

[ece310067-bib-0049] Pintor, L. M. (2007). *The influence of invader traits and community characteristics on the invasion success of an exotic crayfish*. (Doctoral dissertation) University of California.

[ece310067-bib-0050] Pintor, L. M. , & Sih, A. (2009). Differences in growth and foraging behavior of native and introduced populations of an invasive crayfish. Biological Invasions, 11(8), 1895–1902. 10.1007/s10530-008-9367-2

[ece310067-bib-0051] R Core Team . (2022). R: A language and environment for statistical computing. R Foundation for Statistical Computing https://www.R‐project.org/

[ece310067-bib-0052] Reznick, D. (1982). The impact of predation on life history evolution in Trinidadian guppies: Genetic basis of observed life history patterns. Evolution, 36(6), 1236–1250. 10.2307/2408156 28563575

[ece310067-bib-0053] Reznick, D. (1985). Costs of reproduction: An evaluation of the empirical evidence. Oikos, 44(2), 257–267. 10.2307/3544698

[ece310067-bib-0054] Sand, H. (1996). Life history patterns in female moose (*Alces alces*): The relationship between age, body size, fecundity and environmental conditions. Oecologia, 106, 212–220. 10.1007/BF00328601 28307646

[ece310067-bib-0055] Snedden, W. A. (1990). Determinants of male mating success in the temperate crayfish *Orconectes rusticus*: Chela size and sperm competition. Behaviour, 115(1–2), 100–113. 10.1163/156853990X00301

[ece310067-bib-0056] Snyder, W. E. , & Evans, E. W. (2006). Ecological effects of invasive arthropod generalist predators. Annual Review of Ecology, Evolution, and Systematics, 37, 95–122. 10.1146/annurev.ecolsys.37.091305.110107

[ece310067-bib-0057] Stearns, S. C. (1989). Trade‐offs in life‐history evolution. Functional Ecology, 3(3), 259–268. 10.2307/2389364

[ece310067-bib-0058] Stearns, S. C. (1992). The evolution of life histories. Oxford University Press.

[ece310067-bib-0059] Stein, R. A. (1976). Sexual dimorphism in crayfish chelae: Functional significance linked to reproductive activities. Canadian Journal of Zoology, 54(2), 220–227. 10.1139/z76-024

[ece310067-bib-0060] Stein, R. A. , Murphy, M. L. , & Magnuson, J. J. (1977). External morphological changes associated with sexual maturity in the crayfish (*Orconectes propinquus*). The American Midland Naturalist, 97(2), 495–502. 10.2307/2425115

[ece310067-bib-0061] Suko, T. (1958). Studies on the development of the crayfish VI. The reproductive cycle. Science Reports of Saitama University (Japan) B, 3, 79–91.

[ece310067-bib-0062] Tierney, A. J. , Gunaratne, C. , Jennison, K. , Monroy, V. , & Donnelly, L. (2008). Behavioral correlates of alternate male forms (form I and form II) in the crayfish *Orconectes rusticus* . Journal of Crustacean Biology, 28(4), 596–600. 10.1651/08-2984.1

[ece310067-bib-0063] Van Noordwijk, A. J. , & de Jong, G. (1986). Acquisition and allocation of resources: Their influence on variation in life history tactics. The American Naturalist, 128(1), 137–142. 10.1086/284547

[ece310067-bib-0064] Weagle, K. V. , & Ozburn, G. W. (1970). Sexual dimorphism in the chela of *Orconectes virilis* (Hagen). Canadian Journal of Zoology, 48(5), 1041–1042. 10.1139/z70-183

[ece310067-bib-0065] Weagle, K. V. , & Ozburn, G. W. (1972). Observations on aspects of the life history of the crayfish, *Orconectes virilis* (Hagen), in northwestern Ontario. Canadian Journal of Zoology, 50(3), 366–370. 10.1139/z72-053

[ece310067-bib-0066] Wenner, A. M. , Fusaro, C. , & Oaten, A. (1974). Size at onset of sexual maturity and growth rate in crustacean populations. Canadian Journal of Zoology, 52(9), 1095–1106. 10.1139/z74-147 4417012

[ece310067-bib-0067] Werner, E. E. (1988). Size, scaling, and the evolution of complex life cycles. In Size‐structured populations (pp. 60–81). Springer. 10.1007/978-3-642-74001-5_6

[ece310067-bib-0068] Werner, E. E. , & Gilliam, J. F. (1984). The ontogenetic niche and species interactions in size‐structured populations. Annual Review of Ecology and Systematics, 15, 393–425. 10.1146/annurev.es.15.110184.002141

[ece310067-bib-0069] Wetzel, J. E. (2002). Form alternation of adult female crayfishes of the genus Orconectes (Decapoda: Cambaridae). The American Midland Naturalist, 147(2), 326–337. 10.1674/0003-0031(2002)147[0326:FAOAFC]2.0.CO;2

[ece310067-bib-0070] Williams, G. C. (1966). Adaptation and natural selection. Princeton University Press.

